# Cross-feeding between cyanobacterium *Synechococcus* and *Escherichia coli* in an artificial autotrophic–heterotrophic coculture system revealed by integrated omics analysis

**DOI:** 10.1186/s13068-022-02163-5

**Published:** 2022-06-22

**Authors:** Jiajia Ma, Taohong Guo, Meijin Ren, Lei Chen, Xinyu Song, Weiwen Zhang

**Affiliations:** 1 Laboratory of Synthetic Microbiology, School of Chemical Engineering & Technology, Tianjin University, Tianjin, 300072 People’s Republic of China; 2 Key Laboratory of Systems Bioengineering (Ministry of Education), Tianjin University, Tianjin, 300072 People’s Republic of China; 3 SynBio Research Platform, Collaborative Innovation Center of Chemical Science and Engineering, Tianjin, 300072 People’s Republic of China; 4 Center for Biosafety Research and Strategy, Tianjin University, Tianjin, 300072 People’s Republic of China; 5 Center for Excellence in Molecular Plant Sciences, Chinese Academy of Sciences, Shanghai, 200032 People’s Republic of China

**Keywords:** Artificial coculture system, Cyanobacteria, Interaction mechanism, Quantitative proteomics, Transcriptomics, Metabolomics

## Abstract

**Background:**

Light-driven consortia, which consist of sucrose-secreting cyanobacteria and heterotrophic species, have attracted considerable attention due to their capability for the sustainable production of valuable chemicals directly from CO_2_. In a previous study, we achieved a one-step conversion of sucrose secreted from cyanobacteria to fine chemicals by constructing an artificial coculture system consisting of sucrose-secreting *Synechococcus elongateus cscB*^+^ and 3-hydroxypropionic acid (3-HP) producing *Escherichia coli* ABKm. Analyses of the coculture system showed that the cyanobacterial cells grew better than their corresponding axenic cultures. To explore the underlying mechanism and to identify the metabolic nodes with the potential to further improve the coculture system, we conducted integrated transcriptomic, proteomic and metabolomic analyses.

**Results:**

We first explored how the relieved oxidative stress affected cyanobacterial cell growth in a coculture system by supplementing additional ascorbic acid to CoBG-11 medium. We found that the cell growth of cyanobacteria was clearly improved with an additional 1 mM ascorbic acid under axenic culture; however, its growth was still slower than that in the coculture system*,* suggesting that the improved growth of *Synechococcus cscB*^+^ may be caused by multiple factors, including reduced oxidative stress. To further explore the cellular responses of cyanobacteria in the system, quantitative transcriptomics, proteomics and metabolomics were applied to *Synechococcus cscB*^+^. Analyses of differentially regulated genes/proteins and the abundance change of metabolites in the photosystems revealed that the photosynthesis of the cocultured *Synechococcus cscB*^+^ was enhanced. The decreased expression of the CO_2_ transporter suggested that the heterotrophic partner in the system might supplement additional CO_2_ to support the cell growth of *Synechococcus cscB*^+^. In addition, the differentially regulated genes and proteins involved in the nitrogen and phosphate assimilation pathways suggested that the supply of phosphate and nitrogen in the Co-BG11 medium might be insufficient.

**Conclusion:**

An artificial coculture system capable of converting CO_2_ to fine chemicals was established and then analysed by integrated omics analysis, which demonstrated that in the coculture system, the relieved oxidative stress and increased CO_2_ availability improved the cell growth of cyanobacteria. In addition, the results also showed that the supply of phosphate and nitrogen in the Co-BG11 medium might be insufficient, which paves a new path towards the optimization of the coculture system in the future. Taken together, these results from the multiple omics analyses provide strong evidence that beneficial interactions can be achieved from cross-feeding and competition between phototrophs and prokaryotic heterotrophs and new guidelines for engineering more intelligent artificial consortia in the future.

**Supplementary Information:**

The online version contains supplementary material available at 10.1186/s13068-022-02163-5.

## Introduction

Cyanobacteria with the capability of producing organic matter from CO_2_ using solar energy have attracted increased attention as environmentally friendly and sustainable “microbial cell factories” for the production of carbohydrate feedstocks to support traditional fermentation processes [1, 2]. For example, sucrose is an easily fermentable feedstock. Several cyanobacterial species are capable of synthesizing and secreting sucrose as an osmolyte under appropriate environmental stimuli, such as osmotic pressure [3], and this process can be sustained over a long period of time and at higher levels than that from plant feedstocks such as sugarcane and beet [4, 5]. However, purification of sucrose from cyanobacterial cultivation supernatant is costly, and the system is easily contaminated, which creates barriers to any scale-up cultivation [6]. In addition, any application of photosynthetic cell factories in scale-up facilities is always restricted by challenges from harsh environments, suggesting that the adaptability and compatibility of cyanobacterial cell factories should be further improved to facilitate industrial-scale biomanufacturing [7]. In recent years, increasing evidence has suggested that the exchange of essential metabolites between microorganisms could be a crucial process that can significantly affect the growth, composition and structural stability of microbial communities in nature [8, 9]. For instance, in aquatic environments, the ecological interaction between photoautotrophic and heterotrophic species is based on cross-feeding and metabolite exchange [10]. To date, multiprotein complexes that cross cell membrane(s) and extracellular vesicles have been evaluated in photoautotrophs for transporting materials from the interior to the exterior of the cell [11], which facilitate the secretion of various chemicals ranging from targeted photosynthetic intermediates, such as glycolate, osmolytes and fatty acids, and extracellular polymeric substances, to the products of cell lysis, including sugars, proteins, lipids and nucleic acids [12, 13]. These organic compounds could support the cell growth of the heterotrophic species. In addition, heterotrophic species are also thought to provide essential micronutrients, such as vitamins and amino acids, which are beneficial to maintain high photosynthetic productivity [9]. In addition, some positive effects on promoting cell growth were also observed, which may be attributed to the decreased oxidative stress by heterotrophs through reactive oxygen species (ROS) scavenging [14, 15]. Inspired by the symbiotic system commonly found in nature, increasing efforts have been made in recent years to design artificial routes of metabolite interchange to construct new symbiotic systems with high efficiency and stability [16, 17].

Light-driven artificial consortia consisting of sucrose-secreting cyanobacteria and heterotrophic species have recently attracted significant attention due to the potential to be alternatives for the utilization of sucrose secreted from cyanobacteria [18]. For example, Ducat et al. constructed a coculture system consisting of the cyanobacterium *Synechococcus elongatus* PCC 7942 (hereafter *Synechococcus* 7942) and the heterotrophic bacterium *Halomonas boliviensis*, in which the growth of *H. boliviensis* was supported by sucrose produced by *S. elongatus* 7942 [19]. In another study, Li et al. designed a coculture system with sucrose-secreting *S. elongatus* 7942 and three different yeasts to mimic lichen and to study the interaction between autotrophic and heterotrophic strains [14]. More recently, Liu et al. constructed a coculture system composed of *S. elongatus* 7942 and *E. coli* to produce isoprene. The obtained results showed that the fermentation time of the coculture system was extended from 100 to 400 h by adjusting the inoculation ratio between *S. elongatus* 7942 and *E. coli*. Moreover, in this system, the production of isoprene was increased sevenfold to 0.4 g/L compared to the axenic culture of *E. coli* [20]. Zhang et al. constructed a microbial consortium consisting of the fast-growing cyanobacterium *Synechococcus elongatus* UTEX 2973 (hereafter *Synechococcus* 2973), which is capable of growing under high light and temperature conditions [21], as well as *E. coli*, to sequentially produce sucrose and then the platform chemical 3-hydroxypropionic acid (3-HP) from CO_2_ [22]. All abovementioned studies suggested that a light-driven coculture system could be a promising strategy for future CO_2_-based biomanufacturing and could also be an attractive approach for converting sucrose into value-added chemicals.

To construct light-driven coculture systems with high efficiency, it is necessary to fully understand the metabolic mechanism underlying the interaction between autotrophs and heterotrophs. Although several previous studies have shown that cyanobacterial cell growth could be improved in coculture systems [14], the mechanism has yet to be determined. Moreover, while it is fully expected that the mechanism involves more than just a single gene or even a single metabolic node, to date, only few studies have utilized global-based omics techniques to explore the interaction mechanism [23–25]. Due to the complexity of the coculture structure, the challenge of studying the interaction mechanism is also increased. Integrated omics analysis could be a good approach to obtain a “panorama” of cells in coculture systems and reveal novel insights into the biological mechanism ^26^. For example, Amin et al. analysed the signalling and interaction between diatoms and associated bacteria through integrated metabolite and transcriptomic analysis, in which tryptophan and indole-3-acetic acid were determined to be the key signalling molecules involved in the complex exchange of nutrients [27], demonstrating that the approach of integrated transcriptomics, proteomics and metabolomics should be adopted to explore microbial interactions in coculture systems.

In our previous study, Zhang et al. [22] constructed an artificial coculture system that was able to produce ~ 68.29 mg/L 3-HP in 7 days. However, how to improve its productivity and stability remains challenging. In this study, an integrated proteomics, transcriptomics and metabolomics approach was employed to analyse the metabolic responses of cyanobacteria to the heterotrophic partner in the artificial coculture system, which will be valuable for understanding the underlying mechanisms of improving the cell growth of cyanobacteria. Additionally, multiple omics analyses also revealed the potential metabolic nodes that have the potential to improve the efficiency and stability of the coculture system.

## Materials and methods

### Strains and culture conditions

The sucrose-secreting strain *Synechococcus cscB*^+^ (derived from *Synechococcus elongatus* UTEX 2973) and the sucrose-utilizing and 3-HP-producing *E. coli* ABKm strain reported in our previous study were used to construct a coculture system [22]. *Synechococcus cscB*^+^ was cultivated under 100 μmol photons m^−2^ s^−1^ in an illuminating shaking incubator (HNYC-202 T, Honour, Tianjin, China) at 130 rpm and 37 °C or on BG-11 agar plates in an incubator (SPX- 250B-G, Boxun, Shanghai, China) [28]. The *E. coli* ABKm strain was grown on LB medium or agar plates with appropriate antibiotics added to maintain plasmids at 37 °C in a shaking incubator (HNY-100B, Honour, Tianjin, China) at 200 rpm or in an incubator, respectively. Coculture medium (hereafter CoBG-11) was used to construct a coculture system according to a previous study [22], in which 150 mM NaCl, 4 mM NH_4_Cl and 3 g/L 2-[[1,3-dihydroxy-2-(hydroxymethyl) propan-2-yl] amino] ethanesulfonic acid (TES) were supplemented into the BG-11 medium. The pH value was adjusted with NaOH to 8.3.

For construction of the coculture system, exponential-phase *Synechococcus cscB*^+^ (OD_750_≈1.0) was collected and inoculated into 25 mL of CoBG-11 and grown at 30 °C for 48 h to an OD_750_ of 0.5. *E. coli* was cultivated in CoBG-11 with 1 g/L sucrose for 48 h, and then the cells were collected and resuspended in deionized water and inoculated into the 25 mL *Synechococcus cscB*^+^ culture grown on CoBG-11. To separate the two species in the coculture system, a dialysis bag (diameter is 36 mm, molecular weight cut-off is 14 kDa, biosharp, Hefei, China) was used. The *E. coli* ABKm was incubated in the dialysis bag, while *Synechococcus cscB*^+^ was incubated outside in the flask (Additional file 2: Figure S1). The pre-treatment of dialysis bags was performed according to a previous study with some modifications [29]. Briefly, the dialysis bag was cut into small pieces of appropriate length (approximately 10 cm), which were boiled for 10 min with a large volume of 1 mmol/L EDTA (pH 8.0). Then, the dialysis bags were boiled twice with distilled water for 10 min each. The prepared dialysis bag was autoclave-sterilized before use.

The cell density was measured at OD_750_ using a UV-1750 spectrophotometer (Shimadzu, Kyoto, Japan). The co-cultivated *Synechococcus cscB*^+^ was counted by a haemocytometer under a microscope (BX43, Olympus, Shinjuku, Tokyo, Japan) after serial dilution.

### Determination of H_2_O_2_ and ROS concentrations

H_2_O_2_ Quantitative Assay Kit (Water-Compatible) (Sangon Biotech, Shanghai, China) was used to analyse the content of H_2_O_2_ in the medium supernatant. Under acidic conditions, H_2_O_2_ oxidizes Fe^2+^ ions into Fe^3+^ ions, which then combine with dye molecules to form an Fe^3+^-dye complex. The formed complex has a maximum absorption wavelength of 560 nm (or 595 nm), and the absorption value is proportional to the concentration of H_2_O_2_, which was detected by a spectrophotometer (Thermo Fisher Scientific Oy, Vantaa, Finland).

For ROS measurement, cell samples (1 OD750 unit) were harvested and resuspended with BG11. ROS content was measured using a Reactive Oxygen Species Assay Kit (Beyotime, Shanghai, China) following the manufacturer’s protocol. Briefly, the 1 nonfluorescent probe DCFH-DA was finally oxidized by intracellular ROS or other peroxide fluorescent DCF. The DCF was detected using an F-2700 fluorescence spectrophotometer (Hitachi, Japan) at EX 488 nm and EM 525 nm.

### Transcriptomic analysis of cyanobacterial responses to *E. coli* in the coculture system

Considering the characteristics of transcriptomics technology and the accuracy of transcriptomics data, dialysis bags were used to separate cyanobacteria and *E. coli* to construct the coculture system. For transcriptomic analysis, 5 OD_750_ of cocultured and ascorbic acid-treated axenic *Synechococcus cscB*^+^ were collected for extracting RNA samples; meanwhile, the same amount of *Synechococcus cscB*^+^ cultivated under axenic conditions was used as a control. Transcriptomic analysis was conducted by GENEWIZ (Suzhou, China). There were three biological replicates for each sample, and two statistical parameters, fold change > 1.5 and *Q*-value (fdr or padj) ≤ 0.05, were used to determine differentially regulated genes.

### Quantitative proteomics analysis of cyanobacterial responses to *E. coli* in a coculture system

The same weight of 4-day cocultured strains was sampled for proteome analysis. The samples were enzymatically digested by trypsin, followed by isobaric tags for relative and absolute quantification (iTRAQ). The samples were analysed by liquid chromatography–tandem mass spectrometry (LC–MS/MS). Axenic *Synechococcus* 2973 cells with the same incubation time were used as a control. The technical service and quantitative proteomics data were provided by BGI (Shenzhen, China). Three biological replicates for each sample were used. In the case of unmatched biological replicates, two statistical parameters, fold change > 1.2 (the average ratio of the nine comparison groups) and *P*-value < 0.05 (t-test of nine comparison groups), were used to screen differentially regulated proteins.

### Correlation analyses between transcriptome and proteome

For correlation analyses, the genes with a 1.5-fold difference in transcriptome data and the proteins with a fold change value of 1.2 in the proteome were extracted, respectively, and then the corresponding difference multiple and significance information (P-value) were obtained. The data were imported into Linux to construct the database, and the differential protein data were used as query sequences for BLAST comparison. The results with a 90% greater comparison ratio were extracted from the obtained comparison results, and the corresponding information of genes and proteins was extracted.

### Quantitative real-time PCR analysis

For RNA extraction, the 2 OD_750_ cells of *Synechococcus cscB*^+^ under axenic culture and coculture were collected and centrifuged at 7830 rpm and 4 ℃ for 5 min. Total RNA samples were extracted using a Direct-zol™ RNA Miniprep kit (Zym Research, CA, USA) according to the manufacturer’s instructions and then reverse transcribed as cDNA templates using HiScript^®^ II Q RT SuperMix for qPCR (+ gDNA wiper) reagent (Vazyme, China). The quantitative real-time PCR (qPCR) reactions were performed according to the previously described methods [30]. Briefly, the 10-μL reaction system was composed of 5 μL of 2 × ChamQ Universal SYBR qPCR Master Mix (Vazyme, China), 3 μL of RNase-free H_2_O, 0.5 μL (5 μM) of each upstream primer and downstream primer and 1 μL of appropriately diluted cDNA template. The relative changes in gene expression from the qPCR experiment could be analysed using the 2^−ΔΔCT^ method [31].

### LC–MS-based metabolomics analysis

Liquid chromatography–mass spectrometry (LC–MS)-based targeted metabolomics was performed according to the previously described protocol [30]. Cells (5 OD730 unit) were harvested at 48 h via centrifugation at 7380 rpm for 5 min at 4 °C (Eppendorf 5430R), quenched, and rapidly extracted with 900 μL of 80:20 methanol/water (v/v; −80 °C pretreated) and then frozen in liquid nitrogen. Intracellular metabolites were extracted via three freeze/thaw cycles. The aforementioned extraction process was repeated with another 500 μL of 80:20 methanol/water (v/v). The supernatant was combined and filtered through a 0.22-μm syringe filter. The solvents were removed using a vacuum concentrator system (ZLS-1, Hunan, China), and 100 μL of ddH2O was added and mixed well. LC–MS analysis was conducted using an Agilent 1260 series binary HPLC system equipped with a Synergi Hydro-RP (C18) 150 mm × 2.0 mm ID, 4 μm 80 Å particle column (Phenomenex, Torrance, CA, USA.), and an Agilent 6410 triple quadrupole mass analyser equipped with an electrospray ionization (ESI) source. Data were acquired using the Agilent Mass Hunter work-station LC/QQQ acquisition software (version B.04.01), and chromatographic peaks were subsequently integrated via Agilent Qualitative Analysis software (version B.04.00). All metabolomic profiling data were first normalized by the internal control and the cell numbers of the samples. Each condition analysis consisted of four biological replicates and three technical replicates.

## Results and discussion

### Effects of oxidative stress on cyanobacterial cell growth in the coculture system

**Fig. 1:**
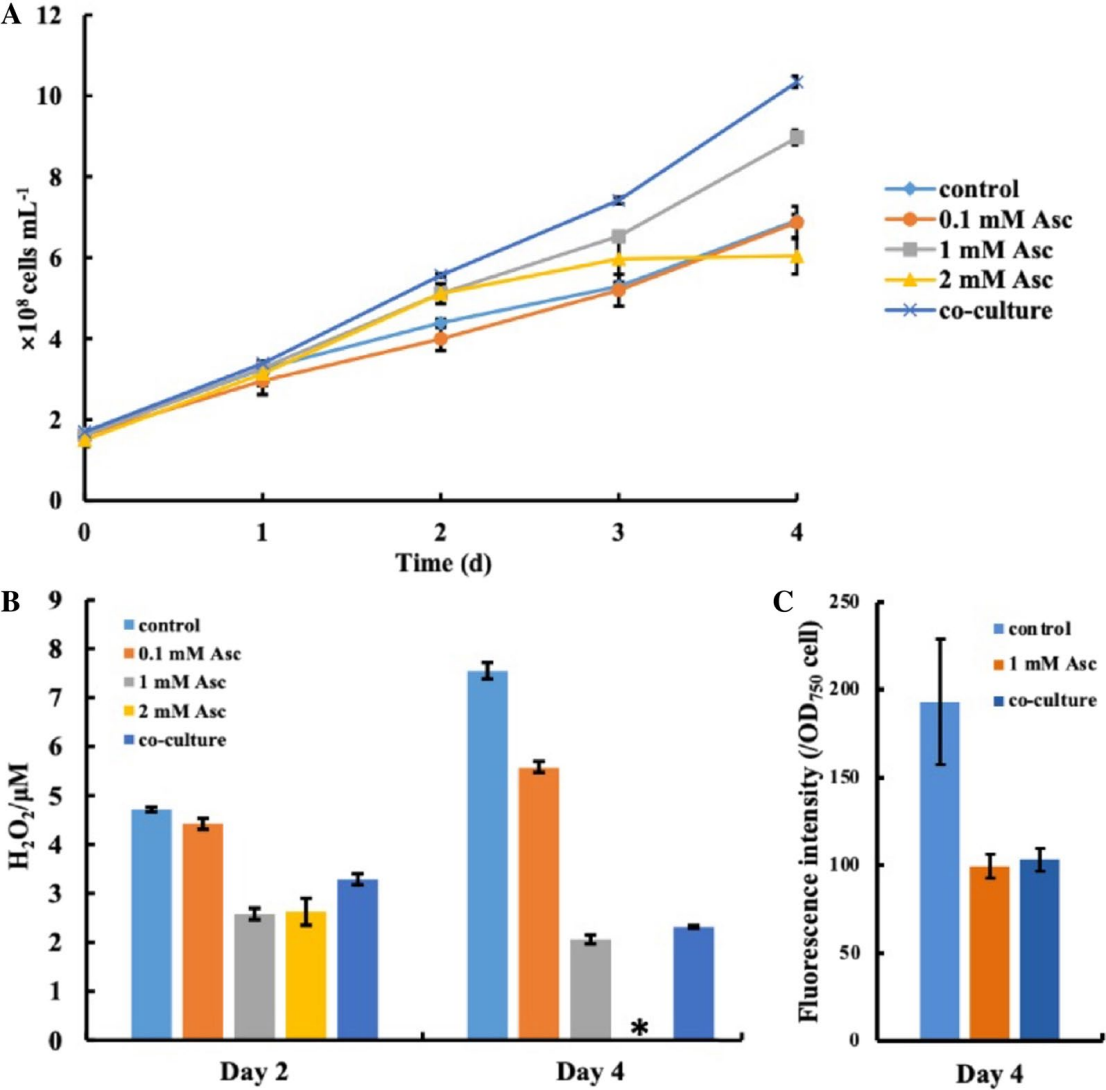
Analysis of the effect of quenching ROS on cultivated cyanobacterial cell growth by adding ascorbic acid. Cell growth curves of *Synechococcus cscB*^+^ (**A**), H_2_O_2_ content (**B**) and ROS level (**C**) in the coculture system and axenic culture with additional ascorbic acid. The coculture system were cultivated in coculture medium (named CoBG-11) under a light intensity of approximately 100 μmol photons m^−2^ s^−1^ in an illuminating shaking incubator at 150 rpm and 30 °C. CoBG-11 medium was designed based on BG-11 medium and optimized for *E. coli* growth by supplementing with 150 mM NaCl, 4 mM NH_4_Cl and 3 g/L 2-[[1,3-dihydroxy-2-(hydroxymethyl) propan-2-yl] amino] ethanesulfonic acid (TES). NH_4_Cl were used to maintain the cell survival of *E. coli*, and NaCl was used as a stress inducer for sucrose accumulation in *Synechococcus* 2973

With the potential to react with biomolecules, including nucleic acids, proteins and lipids, ROS can cause oxidative stress in cells, leading to cellular damage [32]. Previous studies pointed out the possibility that heterotrophic bacteria can reduce oxidative stress in the medium to improve the cell growth of cyanobacteria in a coculture system, eventually enhancing the stability of the coculture system [14, 15]. Ren et al. selected ascorbate as an antioxidant to quench intracellular ROS in the microalgae *Schizochytrium* sp., which has great significance in improving the production of DHA [33]. To test whether the relieved oxidative stress is the determining factor that affects the cell growth of cyanobacteria, different concentrations of ascorbic acid (0.1 mM, 1 mM, 2 mM) were added to axenic cultured *Synechococcus cscB*^+^ in CoBG-11 medium [34–36]. Analysis showed that *Synechococcus cscB*^+^ grew better with additional 1 mM ascorbic acid in CoBG-11 compared with 0.1 mM ascorbic acid. Moreover, the growth rate of the strain in the coculture group (2.39 OD_750_/day) was significantly faster than that in the control (1.27 OD_750_/day) and 1 mM Acs (1.44 OD_750_/day) groups. However, intriguingly, cell growth was inhibited after 3 days of incubation when supplemented with 2 mM ascorbic acid (Fig. 1A). Additionally, as expected, our results showed that the H_2_O_2_ content was significantly decreased when ascorbic acid was added, and on Day 4, the H_2_O_2_ content was not detectable in the condition supplemented with 2 mM ascorbic acid (Fig. 1B). Meanwhile, the intracellular ROS level was also significantly decreased when ascorbic acid was added (Fig. 1C). More importantly, although cyanobacterial growth was clearly improved with additional 1 mM ascorbic acid under axenic culture, cyanobacterial growth was still slower than that in the coculture system*,* suggesting that except for the relieved oxidative stress, other critical factors also contributed to the improved growth of *Synechococcus cscB*^+^ in the coculture system.

### Analysis of the transcriptional profiling of the cyanobacteria in the coculture system

Even though the relieved oxidative stress is beneficial for the cell growth of cyanobacteria in the coculture system, it is also of great significance to decipher the other factors contributing to the faster cell growth as well as the stability of the system. To explore the underlying mechanisms, transcriptomic analysis between axenic culture cyanobacteria (C) and cocultured cyanobacteria (D) was conducted to analyse the transcriptional changes in cyanobacterial responses to the heterotrophic partner in the coculture system. With a cut-off of fold change log_2_ > 1 and a* p* value of statistical significance less than 0.05, we found 78 upregulated genes and 55 downregulated genes in the cocultured cyanobacteria (Additional file 1: Tables S1 and S2). The reliability and accuracy of the transcriptomics data were independently verified by real-time quantitative PCR (qRT-PCR) (Additional file 3: Table S3). Moreover, the correlation coefficient *R*^2^ was 0.9086 (Additional file 2: Figure S2), indicating that the transcriptomics data collected in this study are of very high accuracy. Transcriptomics analysis showed that the differentially expressed genes can be affiliated with most of the essential metabolic modules (Additional file 3: Figure S3), suggesting that the metabolic network of the cyanobacteria was reshaped in the coculture system.

Of them, significant decreases in transcripts of oxidative stress-related genes were found in co-cultivated *Synechococcus cscB*^+^, including high light-inducible protein M744_11065 (Hli), peroxiredoxin Q protein M744_03995 (Tpx-Q), damage-inducible protein M744_11810 (DinB), flavoprotein (Flv) M744_06055 and M744_09730, indicating possible reduced oxidative stress in co-cultivated *Synechococcus cscB*^+^. High light-induced proteins are critical for the energy dissipation mechanism to resist oxidative stress in cyanobacteria ^37^. He et al. found that the gene expression of 4 *hlis* genes was induced under low temperature, strong light stress and nutrient deficiency conditions in *Synechocystis* sp. PCC 6803 (hereafter *Synechocystis* 6803), and the *hli* knockout mutant strain could not survive under strong light, suggesting that the high light-induced protein may play a photoprotective role [38]. Peroxiredoxin Q proteins are important for maintaining redox homeostasis in several organisms. Tailor et al. found that overexpression of peroxiredoxin Q proteins can protect cyanobacteria from oxidative stresses [39]. Moreover, the expression of *dinB* and *flv* was identified to be upregulated under stress tolerance [40, 41]. Consistent with these results, the downregulation of these genes in the cocultured *Synechococcus cscB*^+^ demonstrated that oxidative stress was relieved in this artificial consortium. In a previous study, Vance et al. found that the phospholipid/cholesterol/gamma-HCH transport system permease protein (MlaE) was downregulated after exposure to a high bisphenol A concentration, which might inhibit phospholipid transport and subsequently alter the spontaneous diffusion of the membrane to eventually cause membrane damage [42]. Interestingly, the relative expression of M744_01095 (*mlaE*) was also increased in cocultured *Synechococcus cscB*^+^, which also suggested that oxidative stress-induced membrane damage was relieved.

**Fig. 2:**
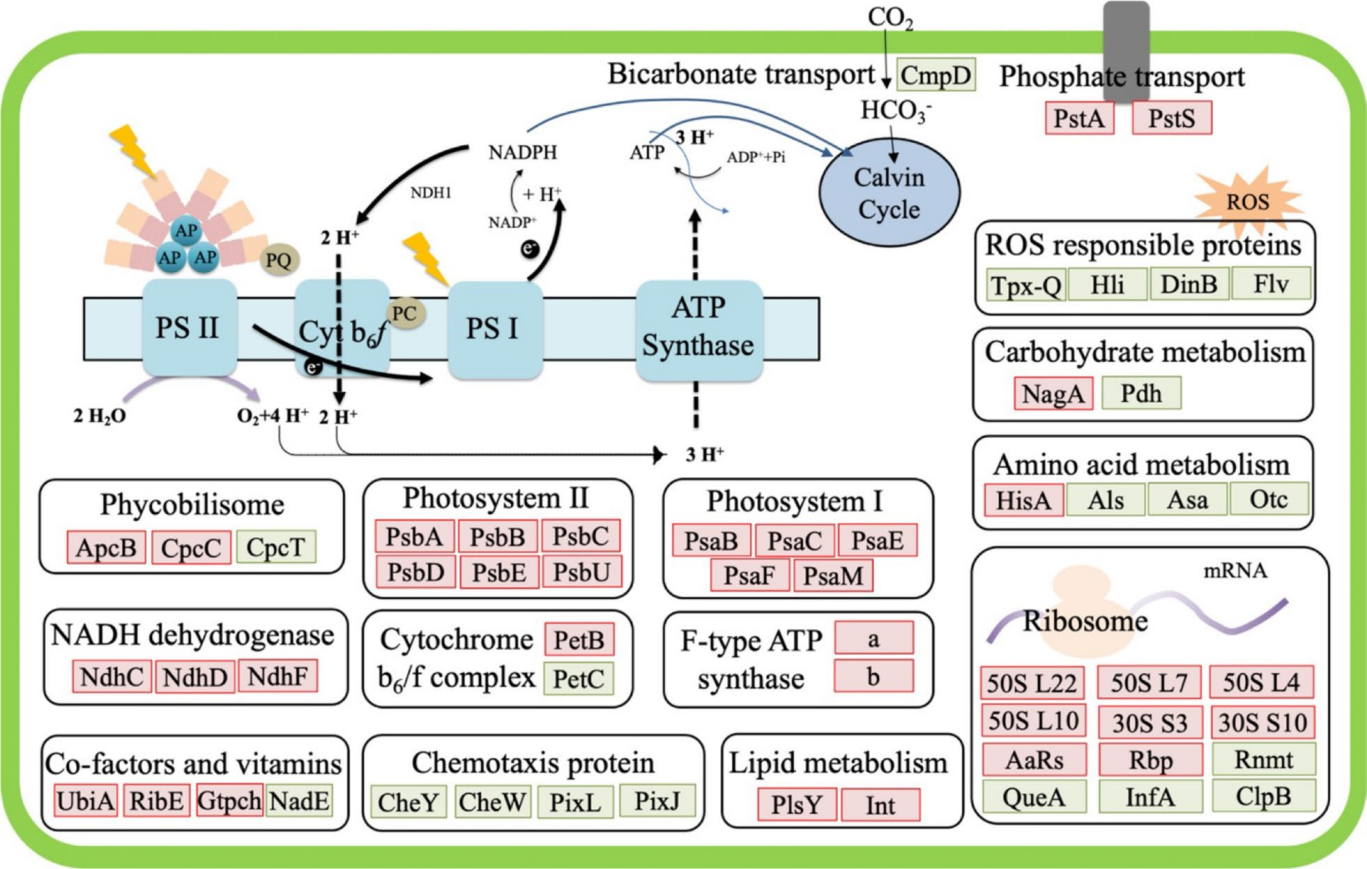
Transcriptomic analysis of *Synechococcus cscB*^+^ under coculture conditions compared with pure culture conditions. With a cut-off of fold change log_2_ > 1 and a* p* value of statistical significance less than 0.05, the differentially expressed genes were selected. Upregulated and downregulated genes are labelled red and green, respectively

Moreover, compared with the axenic strain, ribosomal proteins (*M744_13675*, *M744_13670*, *M744_05205*, *M744_05210*, *M744_12320*, *M744_05185*), tRNA synthetases (*M744_03935*, *M744_05340*) and RNA binding protein (*M744_12800*) were significantly increased (Fig. 2, Additional file 1: Table S1), which indicated that an elevated protein translation and turnover rate might be required to support the high cell growth rate as well as to generate high expression-level heterologous enzymes such as *cscB*^+ 43^. Proteins involved in the photosynthetic apparatus were significantly upregulated, thus suggesting that photosynthesis in the cocultured cyanobacteria was reinforced, which is consistent with the observed improved cell growth (Fig. 1A). A previous study also showed that in cyanobacteria, oxidative stress could impair photosynthesis by downregulating the expression levels of the key genes in the photosystems ^44^. The obvious upregulation of the genes involved in photosynthesis further supported our hypothesis that the improved cell growth of cyanobacteria in the coculture system could be attributed to the relieved oxidative stress (Fig. 1B).

For interactions based on cross-feeding and metabolite exchange, early studies have shown that heterotrophic bacteria can improve the growth of cyanobacteria by scavenging oxygen and ROS and providing metabolites [45]. In this artificial consortium, the consumption of sucrose to support the cell growth of *E. coli* and to produce 3-HP would release CO_2_, which holds the potential to increase the carbon availability for cyanobacteria. However, intriguingly, the *M744_08450* gene (c*mpD* encoding a bicarbonate transporter) was significantly downregulated (Fig. 2, Additional file 1: Table S2). This finding was consistent with previous studies, in which researchers found that high CO_2_ concentrations suppressed the expression levels of CCM-related proteins and the genes encoding bicarbonate transporters (CmpA and CmpC) [46, 47]. Taken together, these results suggested the consumption of sucrose in *E. coli* ABKm, which in turn provided more carbon dioxide to support the growth of cyanobacteria. This cross-feeding relationship has been considered a key factor in stabilizing the long-term fermentation process in coculture systems [26]. Phosphorus (P) is an important nutrient central to storing and exchanging energy and information in cells [48]. Since part of the phosphate was used by *E. coli* in the coculture system, the remaining phosphate may be lower for cyanobacteria. The upregulated phosphate transporters, including the permease genes *M744_04015* (*pstS, sphX*) and *M744_04030* (*pstA*), in the cocultured *Synechococcus cscB*^+^ strain suggested the competitive consumption of phosphate between those two strains, which also paved a new way towards the optimization of this system.

### Differential proteomics analysis of ***Synechococcus cscB***.^+^ between the coculture and axenic conditions

The low correlation between mRNA and protein expression, which may be caused by the widespread posttranscriptional regulation mechanism, has been found and well discussed in previous studies [49, 50]. For example, Nie et al. found that the correlation of mRNA expression and protein abundance was affected at a fairly significant level by multiple factors related to translational efficiency [51]. To fully identify the interaction mechanism in the coculture system, quantitative iTRAQ proteomics was conducted to analyse the cell responses of *Synechococcus cscB*^+^ in the coculture system. Three *Synechococcus cscB*^+^ samples from the coculture (D1, D2, D3) and three from the axenic culture (C1, C2, C3) were collected after cultivation for 96 h, and the differential profiles of proteins in *Synechococcus cscB*^+^ were identified by setting comparison groups of D1 vs. C1, D1 vs. C2, D1 vs. C3, D2 vs. C1, D2 vs. C2, D2 vs. C3, D3 vs. C1, D3 vs. C2, D3 vs. C3. The proteomic analyses identified a total of 914,635 spectra, among which 21,603 unique spectra met the 1% FDR filter and were matched to 2206 unique proteins in the genome. In addition, good coverage was obtained for a wide MW range for protein (Additional file 4: Figure S4A). Most of the identified proteins had good peptide coverage; ~ 89% of the proteins had more than 10% sequence coverage, and ~ 87% had more than 20% sequence coverage (Additional file 4: Figure S4B). Among the functional categories, the “general function prediction only” was the top detected functional category, representing 13.43% of all identified proteins (Additional file 4: Figure S4C). This result is consistent with the previous finding that approximately 45% of proteins in the cyanobacterial genome are hypothetical proteins [52]. Other frequently detected functional categories included “translation, ribosome structure and biogenesis” (9.42%), “amino acid transport and metabolism” (8.67%), “posttranslational modification, protein turnover, chaperones” (8.51%), “signal transduction mechanism” (7.17%), and “carbohydrate transport and metabolism” (6.51%).

**Fig. 3:**
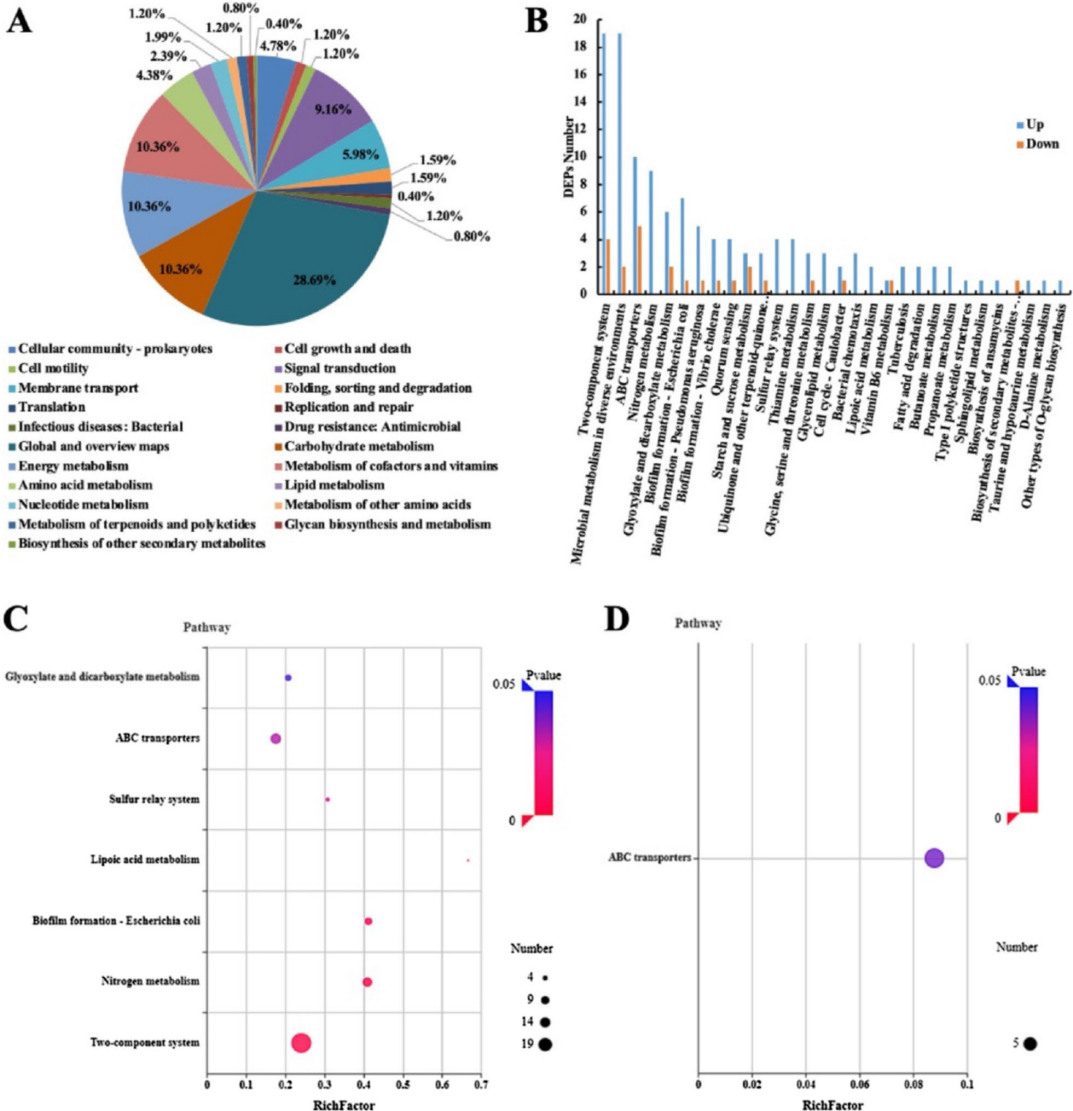
Functional category and enriched pathway items of differentially expressed proteins identified in cocultured *Synechococcus cscB*^+^. **A** Pathway classification distribution of differentially expressed proteins in *Synechococcus cscB*^+^ under coculture compared with pure culture conditions; (**B**) pathway classification statistics of the upregulated and downregulated proteins in *Synechococcus cscB*.^+^ under coculture vs. pure culture conditions; (**C**) enriched pathway items of upregulated differentially expressed proteins; (**D**) enriched pathway items of downregulated differentially expressed proteins

All 251 differentially expressed proteins were divided into 21 secondary branches of pathways based on the KEGG database classification, in which 181 differentially regulated proteins were related to cell metabolism, including energy metabolism (10.36%), metabolism of cofactors and vitamins (10.36%), carbohydrate metabolism (10.36%), and amino acid metabolism (4.38%) (Fig. 3A). The number of up- and down-regulated proteins in each annotated pathway is shown in Fig. 3B. The KEGG enrichment analysis suggested that seven pathways were significantly enriched (*P value* < 0.05) in the upregulated differential proteins, including “two-component system”, “nitrogen metabolism”, and “biofilm formation-*E. coli*”, “lipoic acid metabolism”, “sulfur relay system”, “ABC transporters”, and “glyoxylate and dicarboxylate metabolism” (Fig. 3C), while only “ABC transporters” was significantly enriched (*P*-value < 0.05) in the downregulated differential proteins (Fig. 3D). In addition, a low correlation coefficient (0.0605) was obtained between transcriptomics and proteomics, also indicating that posttranscriptional regulation may play significant roles (Additional file 5: Figure S5).

### Functional analysis of the differential proteome in cocultured ***Synechococcus cscB***^+^

**Fig. 4:**
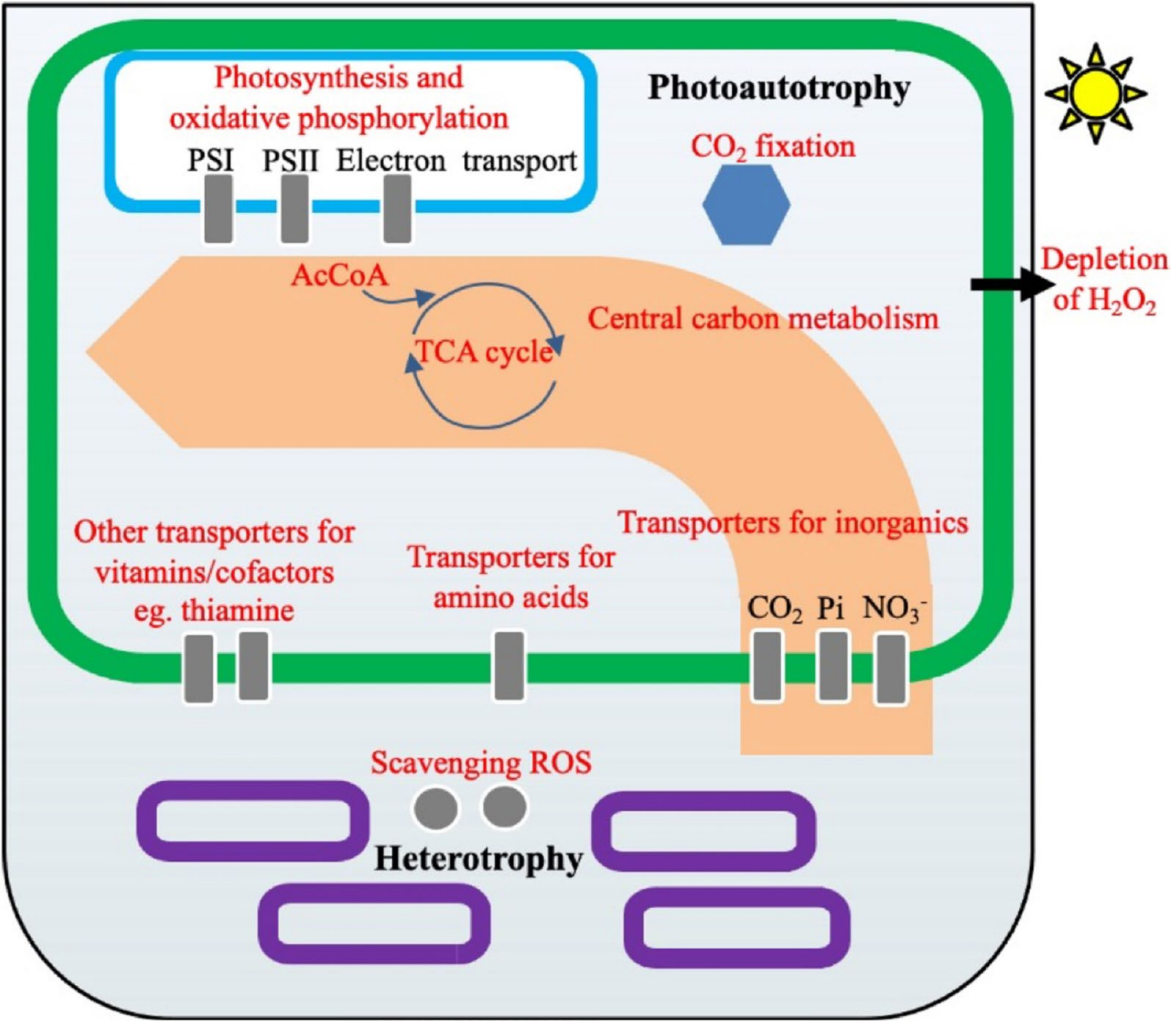
Schematic representation of the cross-feeding process from heterotrophic to photoautotrophic species occurring in the artificial coculture system. The purple boxes represent the heterotrophic bacteria, and the green boxes represent the photoautotrophic bacteria. Abundantly detected processes/pathways/transporters/metabolites are represented in red in the photoautotrophic cell

Although transcriptomics and proteomics are critical to study the interaction mechanisms of the coculture system, the potential incompleteness of these different methods still hinders the full understanding of the biological responses. With complementary characteristics, multiple-omics analysis has been widely applied in deciphering the panorama of metabolic changes in coculture systems [27]. In the cocultured *Synechococcus cscB*^+^ strain, transcriptomic analysis showed that a large fraction (25%) of transcripts involved in photosynthesis and oxidative phosphorylation were significantly upregulated. Consistently, some of the differentially expressed proteins involved in the energy metabolism pathway were also observed in the proteomics data. Specifically, in cocultured *Synechococcus cscB*^+^, proteins involved in energy metabolism, such as ferredoxin (PetF, M744_01325), phycobiliprotein terminal rod linker (CpcD, M744_11425), photosystem II reaction centre H (PsbH, M744_01910), photosystem II D1 protein (PsbA, M744_00850), NAD(P)H-quinone oxidoreductase subunit 4 (NadhD, M744_05920), and NAD(P)H-quinone oxidoreductase subunit 5 (NadhF, M744_01470), were upregulated (Fig. 4, Additional file 1: Table S4), suggesting that photosynthesis may be reinforced [53].

The *cmp* operon (*cmpA*, *cmpB*, *cmpC*, *cmpD*) in *Synechococcus* 7942 has been confirmed to encode a component of the ABC-type HCO^3−^ transporter BCT1, and its transcription was repressed under high CO_2_ conditions [46, 47]. The transcriptomics data showed that *M744_08450* (encoding c*mpD*) was significantly downregulated, while the changes in the other three genes in this operon were not obvious. Interestingly, proteomics analysis showed that M744_08440 (CmpB), M744_08445 (CmpC), and M744_08450 (CmpD) were downregulated (Additional file 1: Table S4), which is consistent with our hypothesis that this repression may be induced by the elevated CO_2_ in the coculture system. The downregulation of this bicarbonate transporter indicated that the concentration of CO_2_ in the coculture system might be higher than that under axenic culture conditions because the degradation of sucrose in *E. coli* ABKm might release CO_2_ into the system, which in turn supported the higher cell growth of *Synechococcus* cscB^+^. Furthermore, as the sucrose metabolism pathway was constructed for *E. coli* W, the extra CO_2_ produced from *E. coli* ABKm from sucrose in coculture system could also be estimated according to a previous publication that CO_2_ production rate in *E. coli* W was 16.31 ± 0.82 mmol gDCW^−1^ h^−1^ [54], or approximately 0.718 g CO_2_ in one hour by 1 g DCW *E. coli* W. Combined with the CO_2_ supplied by flask shaking which is roughly 3–5% [55, 56], the estimated ratio of CO_2_ could be 6.73% (v/v) during cocultivation, which is indeed a high CO_2_ condition for cyanobacteria.

Phosphorus is a vital nutrient for cyanobacterial growth. Those proteins involved in the phosphate transport system, including M744_04030 (PstA), M744_04035 (PstB), M744_04025 (PstC), M744_04020 (PstS) and M744_04015 (SphX), were upregulated by 2.08-, 2.12-, 3.80-, 2.67- and 4.75-fold, respectively (Additional file 1: Table S4), which is also consistent with the transcriptomics data. Moreover, increased transcript levels of *M744_04015* (*sph*X) and *M744_04030* (*pst*A) were also found. Instead of inorganic phosphate, dissolved organic phosphorus is used by cyanobacteria via alkaline phosphatase [57]. Interestingly, two alkaline phosphatases (M744_09635 and M744_11635) in co-cultivated *Synechococcus cscB*^+^ were found to be upregulated by 2.89- and 1.40-fold, respectively, suggesting that cyanobacteria would be able to acquire more organic phosphorus for cell growth during cocultivation conditions [58]. This may be the effect of competition with *E. coli* for phosphate during coculture.

Our proteomics analyses also found that some proteins involved in the different metabolic modules showed differential translation patterns that were not correlated with the transcriptomics data. For instance, increased protein abundance of light-independent protochlorophyllide reductase subunit B (ChlB) (M744_07280), which catalyses the conversion of protochlorophyllide to chlorophyllide *a* [59], was observed (Additional file 1: Table S4), suggesting that the biosynthesis and turnover rate of photosynthetic pigments might be improved during cocultivation. Nitrogen metabolism, either from nitrate or ammonium, governs the turnover of the macromolecules that regulate metabolic pathways, eventually affecting energy production and the carbon skeleton [60]. Through quantitative proteomics analysis, three nitrate/nitrite transport system ATP-binding proteins, M744_10450 (NrtB), M744_10455 (NrtC), and M744_10460 (NrtD), and two ferredoxin-nitrite reductases (M744_10440 and M744_07195) were found to be upregulated in co-cultivated *Synechococcus cscB*^+^, suggesting that nitrite uptake in co-cultivated *Synechococcus cscB*^+^ was enhanced. Ammonium is incorporated into carbon skeletons through glutamine synthetase (M744_02210), which was also found to be upregulated in cocultured *Synechococcus cscB*^+^ (Additional file 1: Table S4). Significant upregulation of the proteins in the nitrogen uptake and assimilation pathways was consistent with the observed higher photosynthesis and better cyanobacterial growth under the coculture condition.

Regarding the relieved oxidative stress in the coculture system, proteomics data also revealed that proteins including Hli protein (M744_01810 and M744_11065) and *fur* family transcription regulator (Fur) (M744_05500 and M744_12665) were downregulated in the co-cultivated *Synechococcus cscB*^+^ strain (Additional file 1: Table S4). Of them, the downregulated transcripts of *M744_11065* were identified and discussed in transcriptomic data. In prokaryotes, Fur, usually as an iron uptake regulator, is responsible for controlling the gene expression of siderophore biosynthesis and iron transport ^61^. In previous studies, it has been observed that the expression of the *fur* gene was drastically upregulated under oxidative stress conditions, suggesting the potential crosstalk between controlling iron homeostasis and defending against ROS [62]. By analysing the proteomics data, more clues were found. For instance, a significant decrease in the protein abundance of Fe^3+^ transporters, including AfuA (M744_05470) and AfuB (M744_09555) [60], was observed from the proteomics data, which indicated that iron transportation in the cocultured *Synechococcus cscB*^+^ strain might be decreased. This downregulation of iron transportation would be consistent with the relieved oxidative stress because iron catalysis is necessary for the Haber–Weiss reaction, which generates the most reactive species ^•^OH among all ROS from H_2_O_2_ and O_2_^•^− [61]. Taken together, the downregulation of these proteins indicated that the heterotrophic partner could relieve oxidative stress, which is consistent with our previous findings that the expression levels of the three types of catalases, including hydroperoxidase I (*kat*G), hydroperoxidase II (*kat*F), and hydroperoxidase III (*kat*E), were significantly induced in *E. coli* under cocultivation conditions [22].

Interestingly, the upregulation of the biosynthesis of several micronutrients was also observed in the proteomics data. For example, cysteine desulfurase (M744_03415), which catalyses the conversion of L-cysteine to L-alanine and sulfur, was upregulated. In this reaction, the released sulfur is then transferred into scaffold protein to assemble Fe–S clusters [62], and these clusters have been shown to participate in electron transfer, iron–sulfur storage, regulation of gene expression, photosynthesis, and enzyme activity in all kingdoms of life [63]. The upregulation of M744_03415 may be beneficial for cyanobacteria to maintain higher cell growth during coculture. Thiamine pyrophosphate (TPP) acts as a cofactor for several enzymes in key cellular metabolic pathways, such as glycolysis, the pentose phosphate pathway and the citric acid cycle (TCA cycle) [64]. Proteomic analysis showed that phosphomethylpyrimidine synthase (ThiC) (M744_11180), an essential enzyme for TPP biosynthesis, was upregulated in cocultured *Synechococcus cscB*^+^, suggesting enhanced central carbon metabolism in *Synechococcus cscB*^+^. In addition, 2-succinyl-5-enolpyruvyl-6-hydroxy-3-cyclohexene-1-carboxylate synthase (MenD, M744_09410), which is involved in the biosynthesis of menaquinone and phylloquinone, was upregulated. Menaquinones play important roles in electron transport and oxidative phosphorylation, and phylloquinone is the secondary electron carrier in photosystem I [65]. Therefore, the upregulation of MenD would be important in improving photosynthesis in the cocultured *Synechococcus cscB*^+^ strain.

### Target LC–MS metabolomics analysis of ***Synechococcus cscB***.^+^ in the coculture system

**Fig. 5:**
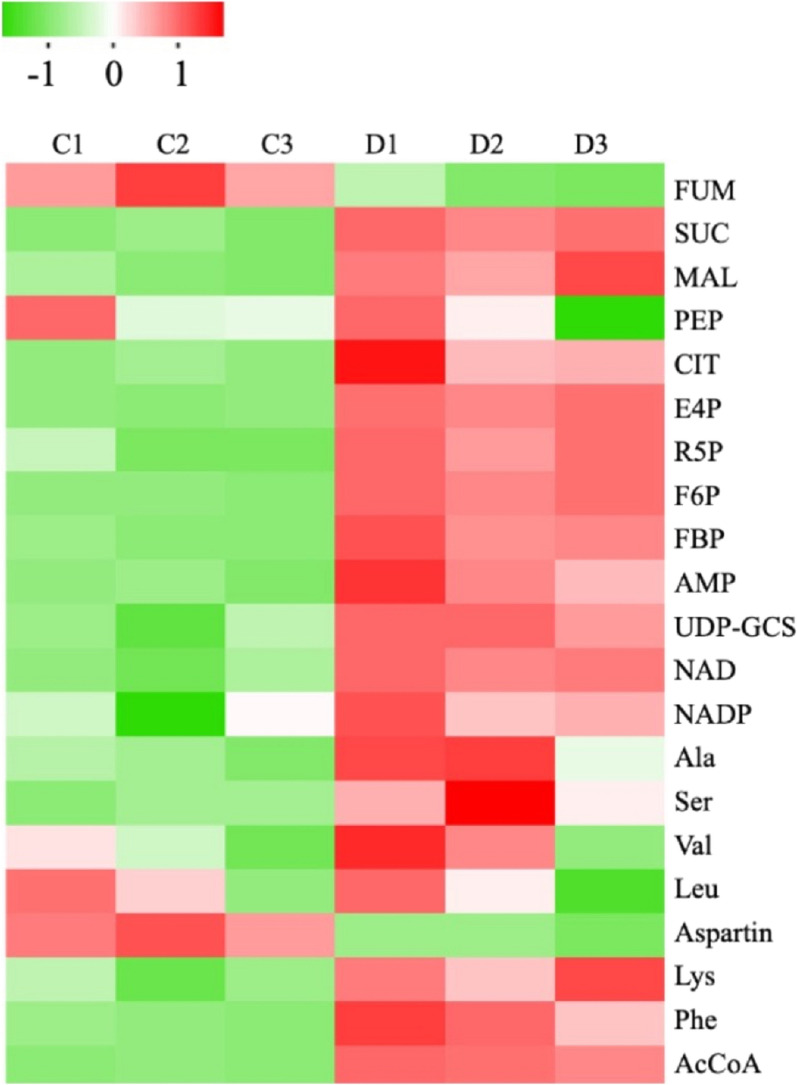
Target metabolomics analysis of *Synechococcus cscB*^+^ under coculture and axenic culture conditions. Heatmaps of metabolomics profiles in *Synechococcus cscB*^+^ under coculture and axenic culture conditions. The cells grown at 48 h under coculture (**D**) and axenic culture conditions (**C**) were harvested and then subjected to analysis. D1, D2 and D3 represent three repeated samples for coculture conditions, and C1, C2 and C3 represent three repeated samples for axenic culture conditions. The colour in the heatmap indicates the log2 transformed ratio of a given metabolite versus the average concentration of the metabolites in all samples

Aside from the transcriptomics and proteomics analyses, targeted metabolomics is useful in quantifying the changes in the intracellular metabolites, which may provide us more insights into the cross-feeding mechanism in the coculture system. Previously, an LC–MS-based metabolomics approach was employed to comparatively analyse cellular metabolism in engineered cyanobacterial strains [66, 67]. In this study, it was applied to compare cocultured and axenic *Synechococcus cscB*^+^. As shown in Fig. 5, 21 metabolites involved in glycolysis, amino acids, and the TCA cycle were detected. Comparative analysis showed that the intracellular contents of FBP, F6P, E4P, R5P and acetyl-CoA were increased. The increased pool size of the metabolites involved in central carbon metabolism indicates that the photosynthesis rate in the cocultured cyanobacteria was enhanced, which might contribute to more available carbon sources produced from the heterotrophic species in the coculture system. Five amino acids, lysine (Lys), serine (Ser), valine (Val), alanine (Ala) and phenylalanine (Phe), were found to be significantly upregulated in coculture cyanobacteria (Fig. 5). A previous study revealed that in symbiotic interactions, autotrophic partners can secrete amino acids to support the cell growth of heterotrophic bacteria [27]. In this coculture system, the concentration of ammonia was approximately 4 mM, which is much lower than that in the standard M9 medium, which may cause nitrogen starvation for the *E. coli* strain. The upregulation of these amino acids would be secreted to compensate for the nitrogen demand of *E. coli*. Even though more evident data are needed, this hypothesis is of great significance to further optimize this coculture system. The abundance of the key metabolite in the TCA cycle, acetyl-CoA, was also increased (Fig. 5). It has been reported that the biosynthesis of acetyl-CoA in *Synechococcus* 2973 is phosphoketolase pathway dependent [68]. Consistently, by analysing the proteomics data, the key enzyme of the phosphoketolase pathway, xylose-5-phosphate/fructose 6-phosphate phosphotransketolase (Xfp), was found to be upregulated in the cocultured *Synechococcus cscB*^+^ strain. Additionally, because UPD-GCS and F6P are the key precursors of the sucrose biosynthetic pathway [69], the significant improvement of these two metabolites was consistent with the high sucrose biosynthesis and secretion in the cocultured cyanobacteria. Because studies have shown that the additional carbon sink would be helpful for increasing photosynthesis in cyanobacteria [70], the increased sucrose sink strength may be another effective factor in enhancing the photosynthesis of cocultured cyanobacteria. Taken together, these multiple omics analyses revealed that the enhanced cell growth of the cocultured *Synechococcus cscB*^+^ strain may result from synergistic effects.

## Conclusions

Stability and long-term productivity are two major parameters that determine the robustness of the artificial consortium. To further improve the performance of the coculture system, an integrated omics analysis was conducted in this study to determine the interaction mechanism between cyanobacterium *Synechococcus* and *E. coli*. Our results showed that the improved cell growth of the cultured *Synechococcus cscB*^+^ strain may result from synergistic effects, including relieved oxidative stress, enhanced CO_2_ availability and increased sucrose sink strength. Multiple omics analyses indicate that many changes we identified tend to be linked with one another at different scales. Moreover, according to the omics analyses, the supply of phosphate and nitrogen in the Co-BG11 medium is not sufficient, which paves a new path towards the optimization of the performance of the coculture system. Taken together, all these results from the multiple omics analyses provide strong evidence that mutual interactions can be obtained from cross-feeding and competition between phototrophs and prokaryotic heterotrophs, which is indispensable for developing more intelligent artificial consortia.

## Supplementary Information

**Additional file 1:** **Table S1.** Upregulated transcripts in co-cultivated *S. elongatus* cscB^+^ compared with those under axenic conditions. **Table S2.** Downregulated transcripts in co-cultivated *S. elongatus* cscB^+^ compared with those under axenic conditions. **Table S3.** Fold change of transcriptomics and qRT-PCR analyses. **Table S4.** Selected differentially expressed proteins associated with cross-feeding and metabolite exchange in co-cultivated *S. elongatus* cscB^+^ compared with those under axenic conditions.

**Additional file 2: Figure S1.** Separation of coculture system based on the dialysis bag. To separate the two species in the coculture system, a dialysis bag was used. The *E. coli* ABKm was incubated in the dialysis bag, while *Synechococcus* *cscB*^+^ was incubated outside in the flask.

**Additional file 3: Figure S2.** Correlation analysis between transcriptomics and qRT-PCR data.

**Additional file 4: Figure S3.** Pathway classification distribution of differentially expressed transcripts in cocultured *Synechococcus cscB*^+^ grown under pure culture conditions. (**A**) Upregulated gene KEGG pathway analysis; (**B**) Downregulated gene KEGG pathway analysis. KEGG, Kyoto Encyclopedia of Genes and Genomes.

**Additional file 5: Figure S4.** Distribution, coverage, and functional category of proteins identified in this study. (**A**) Distribution of proteins identified among different molecular weights; (**B**) Coverage of proteins by the identified peptides; (**C**) Functional category coverage of the proteins identified. Cyanobacterial cells grown for four days under coculture cultivation were harvested for proteome analysis. The same weight of axenic cyanobacterial cells under the same incubation time was used as a control.

**Additional file 6: Figure S5.** Correlation between transcriptomic and proteomic datasets for cocultured *Synechococcus cscB*^+^. The *x*-axis indicates the log2-fold change in genes, and the *y*-axis represents the log2-fold change in proteins. The genes with a 1.5-fold difference in transcriptome data were extracted, and the corresponding difference multiple and significance information (P-value) was obtained. The nine-quadrant diagram reflects the correlation between transcriptomic and proteomic datasets. Quadrant 5 indicates non-differentially expressed genes and proteins with multiple omics; the mRNA in quadrants 3 and 7 showed the same differentially expressed pattern with corresponding proteins; the protein expression abundance in quadrants 1, 2 and 4 represented posttranscriptional or translational level regulation; the protein expression abundance in quadrants 6, 8 and 9 was higher than that in mRNA.

## Data Availability

All data generated or analysed during this study are included in this published article and its additional files.
