# Supplementary material for: Cross-feeding between cyanobacterium Synechococcus and Escherichia coli in an artificial autotrophic–heterotrophic coculture system revealed by integrated omics analysis
Source: Biotechnol Biofuels Bioprod. 2022 Jun 22;15:69. doi: 10.1186/s13068-022-02163-5 (PMC9219151; doi:10.1186/s13068-022-02163-5)
Supplement: Supplementary file 1 — Additional file 1: Table S1. Upregulated transcripts in co-cultivated S. elongatus cscB+ compared with those under axenic conditions. Table S2. Downregulated transcripts in co-cultivated S. elongatus cscB+ compared with those under axenic conditions. Table S3. Fold change of transcriptomics and qRT-PCR analyses. Table S4. Selected differentially expressed proteins associated with cross-feeding and metabolite exchange in co-cultivated S. elongatus cscB+ compared with those under axenic conditions. [file 13068_2022_2163_MOESM1_ESM.docx]

**Supplementary Table S1 Up-regulated transcripts in co-cultivated *S. elongateus* cscB^+^ compared with under axenic condition**

| **Gene ID** | **Description/Gene name** | **Pathway** | **log_2_FoldChange** |
| --- | --- | --- | --- |
| M744_03840 | unknown function |  | 2.52 |
| M744_07860 | unknown function |  | 2.03 |
| M744_07970 | unknown function |  | 1.52 |
| M744_09210 | unknown function |  | 1.50 |
| M744_04090 | unknown function |  | 1.40 |
| M744_00185 | unknown function |  | 1.45 |
| M744_04765 | unknown function |  | 1.39 |
| M744_02825 | unknown function |  | 1.29 |
| M744_01065 | unknown function |  | 1.27 |
| M744_11185 | unknown function |  | 1.19 |
| M744_07700 | unknown function |  | 1.10 |
| M744_10690 | unknown function |  | 1.10 |
| M744_11295 | unknown function |  | 1.98 |
| M744_13015 | unknown function |  | 1.50 |
| M744_13880 | unknown function |  | 1.47 |
| M744_11400 | unknown function |  | 1.40 |
| M744_10945 | unknown function |  | 1.37 |
| M744_02255 | unknown function |  | 1.52 |
| M744_12705 | unknown function |  | 1.42 |
| M744_10925 | unknown function |  | 1.30 |
| M744_04480 | unknown function |  | 1.25 |
| M744_03870 | unknown function |  | 1.24 |
| M744_07370 | unknown function |  | 1.16 |
| M744_07725 | unknown function |  | 1.09 |
| M744_09975 | unknown function |  | 1.08 |
| M744_06420 | unknown function |  | 1.90 |
| M744_08065 | unknown function |  | 1.80 |
| M744_06155 | photosystem I P700 chlorophyll a apoprotein A2, PsaB | Photosynthesis | 1.66 |
| M744_00275 | photosystem I subunit VII, PsaC | Photosynthesis | 1.06 |
| M744_10010 | photosystem I subunit IV, PsaE | Photosynthesis | 1.28 |
| M744_10385 | photosystem I reaction center subunit III, PsaF | Photosynthesis | 1.10 |
| M744_RS06585 | photosystem I reaction center subunit XII, PsaM | Photosynthesis | 1.21 |
| M744_00850 | photosystem II P680 reaction center D1 protein, PsbA | Photosynthesis | 1.85 |
| M744_13305 | photosystem II CP47 chlorophyll apoprotein, PsbB | Photosynthesis | 1.91 |
| M744_13530 | photosystem II reaction center protein CP43, PsbC | Photosynthesis | 1.51 |
| M744_13535 | photosystem II P680 reaction center D2 protein, PsbD | Photosynthesis | 1.27 |
| M744_08300 | photosystem II P680 reaction center D2 protein, PsbD | Photosynthesis | 1.26 |
| M744_10765 | photosystem II cytochrome b559 subunit alpha, PsbE | Photosynthesis | 1.37 |
| M744_07025 | photosystem II PsbU protein, PsbU | Photosynthesis | 1.16 |
| M744_04625 | cytochrome b6, PetB | Photosynthesis | 1.03 |
| M744_01385 | allophycocyanin beta subunit, ApcB | Photosynthesis | 1.25 |
| M744_11430 | phycobilisome rod-core linker polypeptide, CpcC | Photosynthesis | 1.63 |
| M744_11435 | phycobilisome rod-core linker polypeptide, CpcC | Photosynthesis | 1.11 |
| M744_10750 | NAD(P)H-quinone oxidoreductase subunit C, NdhC | Oxidative phosphorylation | 1.41 |
| M744_13795 | NAD(P)H-quinone oxidoreductase subunit 4, NdhD | Oxidative phosphorylation | 1.03 |
| M744_06540 | NAD(P)H-quinone oxidoreductase subunit 4, NdhD | Oxidative phosphorylation | 1.01 |
| M744_05925 | NAD(P)H-quinone oxidoreductase subunit F, NdhF | Oxidative phosphorylation | 1.79 |
| M744_01335 | F0F1 ATP synthase subunit AtpA | Oxidative phosphorylation | 1.47 |
| M744_01360 | F0F1 ATP synthase subunit AtpB | Oxidative phosphorylation | 1.38 |
| M744_05205 | 50S ribosomal protein L22 | Translation | 1.76 |
| M744_13675 | 50S ribosomal protein L7/L12 | Translation | 1.26 |
| M744_05185 | 50S ribosomal protein L4 | Translation | 1.10 |
| M744_13670 | 50S ribosomal protein L10 | Translation | 1.48 |
| M744_05210 | 30S ribosomal protein S3 | Translation | 1.59 |
| M744_12320 | 30S ribosomal protein S10 | Translation | 1.34 |
| M744_03935 | aminoacyl-tRNA biosynthesis, AaRS | Translation | 1.52 |
| M744_05340 | aminoacyl-tRNA biosynthesis, AaRS | Translation | 1.10 |
| M744_12800 | RNA-binding region protein, Rbp | Translation | 1.01 |
| M744_RS06950 | transposase | Genetic information processing | 1.30 |
| M744_13950 | RNase P RNA component class A, RnpB |  | 1.21 |
| M744_00765 | n-acetylglucosamine-6-phosphate deacetylase, NagA | Carbohydrate metabolism | 1.29 |
| M744_08010 | pirin family protein | Signal transduction | 1.38 |
| M744_07055 | transglutaminase, TgA |  | 2.54 |
| M744_04030 | phosphate transport system permease protein, PstA | Transporter | 1.23 |
| M744_04015 | phosphate transport system substrate-binding protein, PstS | Transporter | 2.20 |
| M744_01215 | membrane protein, IctB | Transporter | 1.44 |
| M744_01815 | calcium/sodium antiporter | Transporter | 1.20 |
| M744_08995 | chloride channel protein | Transporter | 1.10 |
| M744_01095 | phospholipid/cholesterol/gamma-HCH transport system permease protein, MlaE | Transporter | 1.13 |
| M744_10350 | chloride channel protein | Transporter | 1.35 |
| M744_02655 | peptidase M50 | Transporter | 1.26 |
| M744_05440 | site-2 protease family protein | Transporter | 1.21 |
| M744_06600 | 4-hydroxybenzoate solanesyltransferase, UbiA | Metabolism of cofactors and vitamins | 1.67 |
| M744_09195 | riboflavin synthase, RibE |  | 1.41 |
| M744_09040 | GTP cyclohydrolase IA, Gtpch |  | 1.06 |
| M744_07325 | 1-(5-phosphoribosyl)-5-[(5-phosphoribosylamino)methylideneamino]imidazole-4-carboxamide isomerase, HisA | Amino acid metabolism | 2.48 |
| M744_06105 | acyl phosphate:glycerol-3-phosphate acyltransferase, PlsY | Lipid metabolism | 2.45 |
| M744_10785 | apolipoprotein N-acyltransferase, Int |  | 2.45 |

**Supplementary Table S2 Down-regulated transcripts in co-cultivated *S. elongateus* cscB^+^ compared with under axenic condition**

| **Gene ID** | **Description/Gene name** | **Pathway** | **log_2_FoldChange** | |
| --- | --- | --- | --- | --- |
| M744_12485 | unknown function |  | -1.63 |  |
| M744_04190 | unknown function |  | -1.35 |  |
| M744_13560 | unknown function |  | -1.20 |  |
| M744_01435 | unknown function |  | -2.06 |  |
| M744_10885 | unknown function |  | -1.62 |  |
| M744_RS14125 | unknown function |  | -1.55 |  |
| M744_09240 | unknown function |  | -1.36 |  |
| M744_00250 | unknown function |  | -1.35 |  |
| M744_01115 | unknown function |  | -1.34 |  |
| M744_03175 | unknown function |  | -1.32 |  |
| M744_01890 | unknown function |  | -1.30 |  |
| M744_00320 | unknown function |  | -1.24 |  |
| M744_05505 | unknown function |  | -1.22 |  |
| M744_09615 | unknown function |  | -1.20 |  |
| M744_04490 | unknown function |  | -1.20 |  |
| M744_07135 | unknown function |  | -1.13 |  |
| M744_03835 | unknown function |  | -1.13 |  |
| M744_03345 | unknown function |  | -1.10 |  |
| M744_00195 | unknown function |  | -1.10 |  |
| M744_03995 | thioredoxin-dependent peroxiredoxin, TPx-Q | Oxidative reducible protein | -1.11 |  |
| M744_11065 | high light inducible protein, Hli | Oxidative reducible protein | -1.10 |  |
| M744_11810 | damage-inducible protein, DinB | Oxidative reducible protein | -1.45 |  |
| M744_06055 | flavoprotein, Flv | Oxidative reducible protein | -1.35 |  |
| M744_09730 | flavoprotein, Flv | Oxidative reducible protein | -1.04 |  |
| M744_06705 | pyruvate dehydrogenase, Pdh | Carbohydrate metabolism | -1.58 |  |
| M744_03660 | ornithine carbamoyltransferase, Otc | Amino acid metabolism | -2.49 |  |
| M744_04075 | acetolactate synthase small subunit, Als | Amino acid metabolism | -1.23 |  |
| M744_05730 | L-aspartate semialdehyde sulfurtransferase, Asa | Amino acid metabolism | -1.02 |  |
| M744_10480 | cytochrome b6-f complex iron-sulfur subunit, PetC | Photosynthesis | -1.24 |  |
| M744_01175 | phycobiliprotein lyase, CpcT | Photosynthesis - antenna proteins | -1.09 |  |
| M744_10320 | RNA methyltransferase, Rnmt | Ribosome biogenesis | -1.52 |  |
| M744_04525 | S-adenosylmethionine:tRNA ribosyltransferase-isomerase, QueA | Transfer RNA biogenesis | -1.37 |  |
| M744_03685 | translation initiation factor IF-1, InfA | Translation factors | -1.13 |  |
| M744_11220 | ATP-dependent chaperone, ClpB | Chaperones and folding catalysts | -1.80 |  |
| M744_02450 | tRNA-Cys | Translation | -1.28 |  |
| M744_12460 | chemotaxis protein, CheY | Bacterial motility proteins | -1.54 |  |
| M744_12455 | chemotaxis protein, CheW | Bacterial motility proteins | -1.42 |  |
| M744_12445 | chemotaxis family, PixL | Bacterial motility proteins | -1.31 |  |
| M744_12450 | methyl-accepting chemotaxis protein, PixJ | Bacterial motility proteins | -1.23 |  |
| M744_00010 | serine protease，HtrA | Protein families: signaling and cellular processes | -1.49 |  |
| M744_08450 | bacitracin ABC transporter ATP-binding protein, CmpD | Transporter | -2.52 |  |
| M744_08660 | molybdate ABC transporter substrate-binding protein, ModA | Transporter | -1.34 |  |
| M744_06915 | hemolysin secretion protein D | Transporter | -1.16 |  |
| M744_10340 | arsenite/tail-anchored protein-transporting ATPase | Transporter | -1.02 |  |
| M744_11255 | metal ABC transporter ATPase | Transporter | -1.36 |  |
| M744_13595 | heavy metal transporter | Transporter | -1.52 |  |
| M744_13590 | circadian clock protein reporter CikA | Circadian clock | -1.03 |  |
| M744_10560 | circadian clock protein KaiB | Circadian clock | -1.21 |  |
| M744_02550 | NAD+ synthase (glutamine-hydrolysing), NadE | Metabolism of cofactors and vitamins | -1.15 |  |
| M744_09255 | iron uptake porin |  | -1.86 |  |
| M744_10365 | cyclic nucleotide-binding domain-containing protein |  | -1.82 |  |
| M744_09995 | replicative DNA helicase, DnaB | Protein families: genetic information processing | -1.14 |  |
| M744_05475 | Crp/Fnr family transcriptional regulator |  | -1.34 |  |
| M744_07365 | tetratricopeptide repeat protein |  | -1.34 |  |
| M744_09590 | glyoxalase |  | -1.18 |  |

**Supplementary Table S3 Foldchange of transcriptomics and qRT-PCR analyses**

| **Genes** | **Description** | **log_2_FoldChange (RNA-seq)** | **log_2_FoldChange (qPCR)** |
| --- | --- | --- | --- |
| *M744_13305* | photosystem II CP47 chlorophyll apoprotein | 1.90±0.15 | 1.39±0.31 |
| *M744_06600* | 4-hydroxybenzoate solanesyltransferase | 1.67±0.33 | 1.73±0.43 |
| *M744_05210* | 30S ribosomal protein S3 | 1.59±0.37 | 1.40±0.44 |
| *M744_00850* | photosystem II q(b) protein | 1.85±0.13 | 2.15±0.03 |
| *M744_01335* | F0F1 ATP synthase subunit alpha | 1.47±0.34 | 1.38±0.24 |
| *M744_00765* | N-acetylglucosamine-6-phosphate deacetylase | 1.29±0.34 | 1.13±0.36 |
| *M744_10010* | photosystem I subunit IV | 1.28±0.39 | 0.72±0.09 |
| *M744_07025* | photosystem II PsbU protein | 1.16±0.29 | 0.84±0.18 |
| *M744_00275* | photosystem I subunit VII | 1.06±0.26 | 1.05±0.51 |
| *M744_06540* | NAD(P)H-quinone oxidoreductase subunit 4 | 1.01±0.15 | 0.61±0.40 |
| *M744_08450* | bacitracin ABC transporter ATP-binding protein | -2.5±0.46 | -3.11±0.18 |
| *M744_03660* | ornithine carbamoyltransferase | -2.49±0.44 | -2.35±0.42 |
| *M744_11220* | ATP-dependent chaperone ClpB | -1.80±0.14 | -1.54±0.54 |
| *M744_06705* | pyruvate dehydrogenase | -1.58±0.33 | -0.51±0.49 |
| *M744_01175* | phycobiliprotein lyase | -1.09±0.35 | -1.25±0.09 |
| *M744_12455* | chemotaxis protein CheW | -1.42±0.27 | -2.17±0.07 |
| *M744_10320* | 23S rRNA (guanine2445-N2)-methyltransferase | -1.52±0.38 | -1.77±0.25 |
| *M744_00010* | serine protease | -1.50±0.16 | -1.87±0.39 |
| *M744_04525* | S-adenosylmethionine:tRNA ribosyltransferase-isomerase | -1.37±0.48 | -0.50±0.28 |
| *M744_11065* | high light inducible protein | -1.10±0.26 | -2.09±0.41 |

**Supplementary Table S4 Selected differentially expressed proteins associated with cross-feeding and metabolite exchange in co-cultivated *S. elongateus* cscB^+^ compared with under axenic condition**

| **Protein** | **Description** | **Mean Ratio** |
| --- | --- | --- |
| M744_01325 | ferredoxin | 1.31 |
| M744_11425 | phycobiliproteins terminal rod linker | 1.28 |
| M744_01910 | photosystem II reaction center H | 1.24 |
| M744_00850 | photosystem II D1 protein | 1.23 |
| M744_05920 | NAD(P)H-quinone oxidoreductase subunit 4 | 1.24 |
| M744_01470 | NAD(P)H-quinone oxidoreductase subunit 5 | 1.21 |
| M744_07280 | light-independent prochlorophyll reductase subunit B | 1.31 |
| M744_05990 | xylulose-5-phosphate/fructose-6-phosphate phosphoketolase | 1.34 |
| M744_04340 | pyruvate-ferredoxin/flavodoxin oxidoreductase | 1.28 |
| M744_10450 | nitrate/nitrite transport system permease protein | 4.33 |
| M744_10460 | nitrate/nitrite transport system permease protein | 1.37 |
| M744_10440 | ferredoxin-nitrite reductases | 1.95 |
| M744_07195 | ferredoxin-nitrite reductase | 1.59 |
| M744_02210 | glutamine synthetase | 1.23 |
| M744_04035 | phosphate transport protein PstB | 2.12 |
| M744_04030 | phosphate transport protein PstA | 2.08 |
| M744_04025 | phosphate transport protein PstC | 3.8 |
| M744_04020 | phosphate transport protein PstS | 2.67 |
| M744_04015 | phosphate transport protein SphX | 4.75 |
| M744_09635 | alkaline phosphatases | 2.89 |
| M744_11635 | alkaline phosphatases | 1.4 |
| M744_05990 | xylulose-5-phosphate/fructose-6-phosphate phosphoketolase | 1.34 |
| M744_04340 | pyruvate-flavodoxin oxidoreductase | 1.28 |
| M744_03415 | cysteine desulfurase | 1.25 |
| M744_11180 | phosphomethylpyrimidine synthase (thiC) | 1.28 |
| M744_09410 | 2-succinyl-5-enolpyruvyl-6-hydroxy-3-cyclohexene-1-carboxylate synthase (menD) | 1.24 |
| M744_01325 | ferredoxin | 1.31 |
| M744_11425 | phycobiliproteins terminal rod linker | 1.28 |
| M744_01910 | photosystem II reaction center H | 1.24 |
| M744_08440 | bicarbonate transporters | -0.75 |
| M744_08445 | bicarbonate transporters | -0.8 |
| M744_08450 | bicarbonate transporters | -0.83 |
| M744_05470 | Fe^3+^ transporter | -0.72 |
| M744_09555 | Fe^3+^ transporter | -0.76 |
| M744_01810 | Hight light inducible protein | -0.75 |
| M744_11065 | Hight light inducible protein | -0.83 |
| M744_05500 | Fur family transcription regulator | -0.55 |
| M744_12665 | Fur family transcription regulator | -0.79 |
| M744_10930 | monothiol glutaredoxin | -0.78 |
