# Supplementary figures and images for: Cross-feeding between cyanobacterium Synechococcus and Escherichia coli in an artificial autotrophic–heterotrophic coculture system revealed by integrated omics analysis
Source: Biotechnol Biofuels Bioprod. 2022 Jun 22;15:69. doi: 10.1186/s13068-022-02163-5 (PMC9219151; doi:10.1186/s13068-022-02163-5)

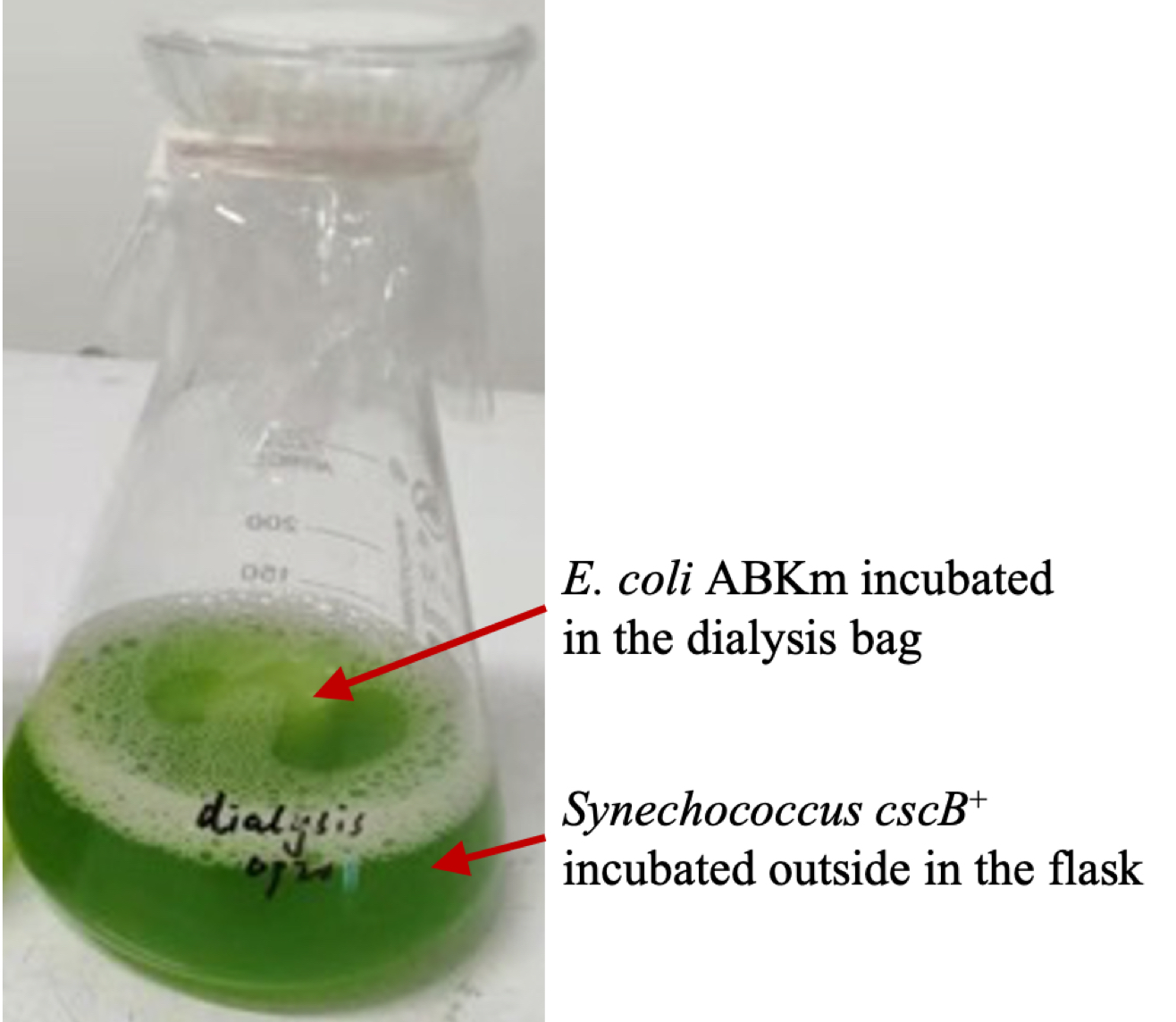

Supplement: Supplementary file 2 — Additional file 2: Figure S1. Separation of coculture system based on the dialysis bag. To separate the two species in the coculture system, a dialysis bag was used. The E. coli ABKm was incubated in the dialysis bag, while Synechococcus cscB+ was incubated outside in the flask. [file 13068_2022_2163_MOESM2_ESM.jpg]

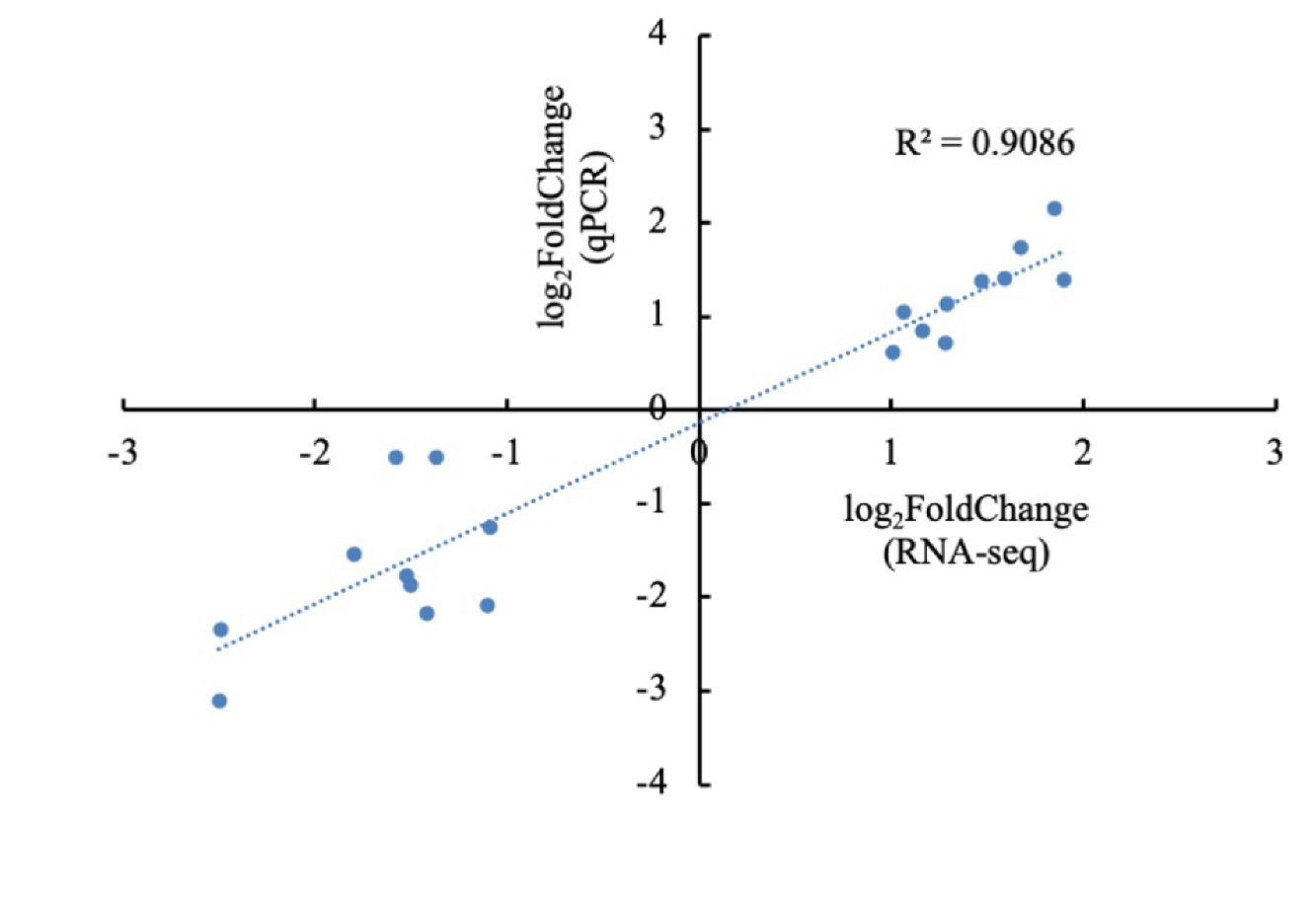

Supplement: Supplementary file 3 — Additional file 3: Figure S2. Correlation analysis between transcriptomics and qRT-PCR data. [file 13068_2022_2163_MOESM3_ESM.jpg]

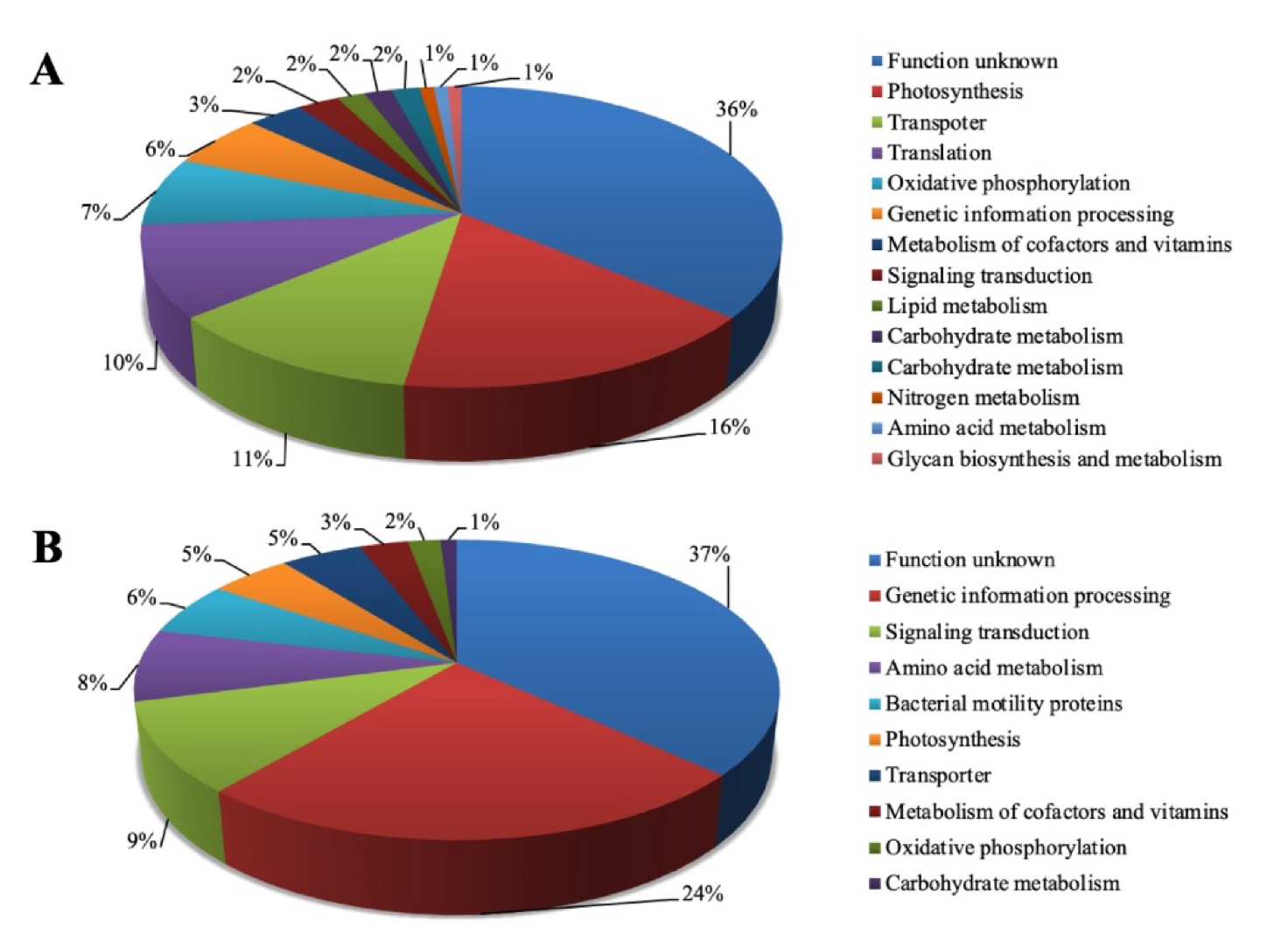

Supplement: Supplementary file 4 — Additional file 4: Figure S3. Pathway classification distribution of differentially expressed transcripts in cocultured Synechococcus cscB+ grown under pure culture conditions. (A) Upregulated gene KEGG pathway analysis; (B) Downregulated gene KEGG pathway analysis. KEGG, Kyoto Encyclopedia of Genes and Genomes. [file 13068_2022_2163_MOESM4_ESM.jpg]

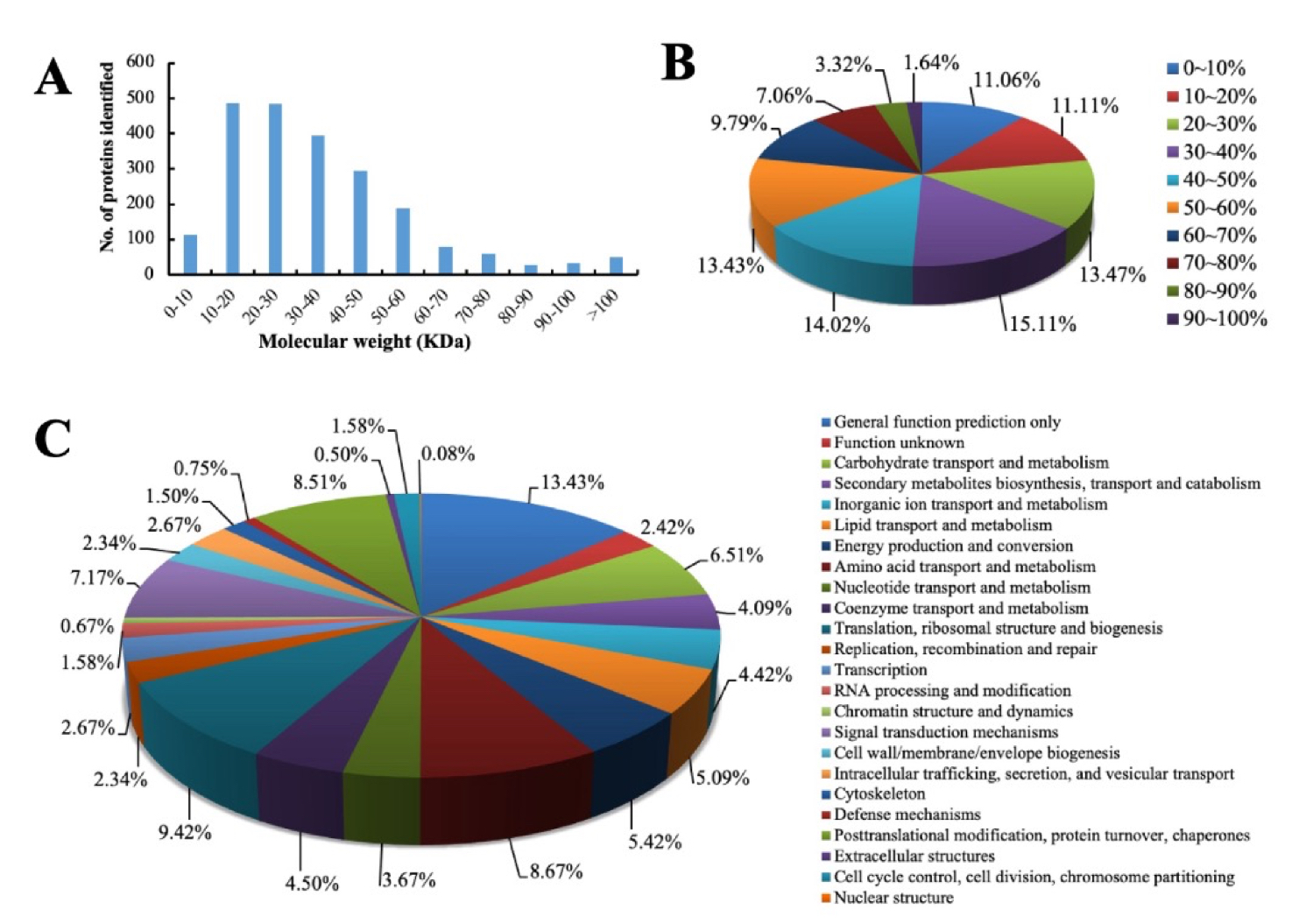

Supplement: Supplementary file 5 — Additional file 5: Figure S4. Distribution, coverage, and functional category of proteins identified in this study. (A) Distribution of proteins identified among different molecular weights; (B) Coverage of proteins by the identified peptides; (C) Functional category coverage of the proteins identified. Cyanobacterial cells grown for four days under coculture cultivation were harvested for proteome analysis. The same weight of axenic cyanobacterial cells under the same incubation time was used as a control. [file 13068_2022_2163_MOESM5_ESM.jpg]

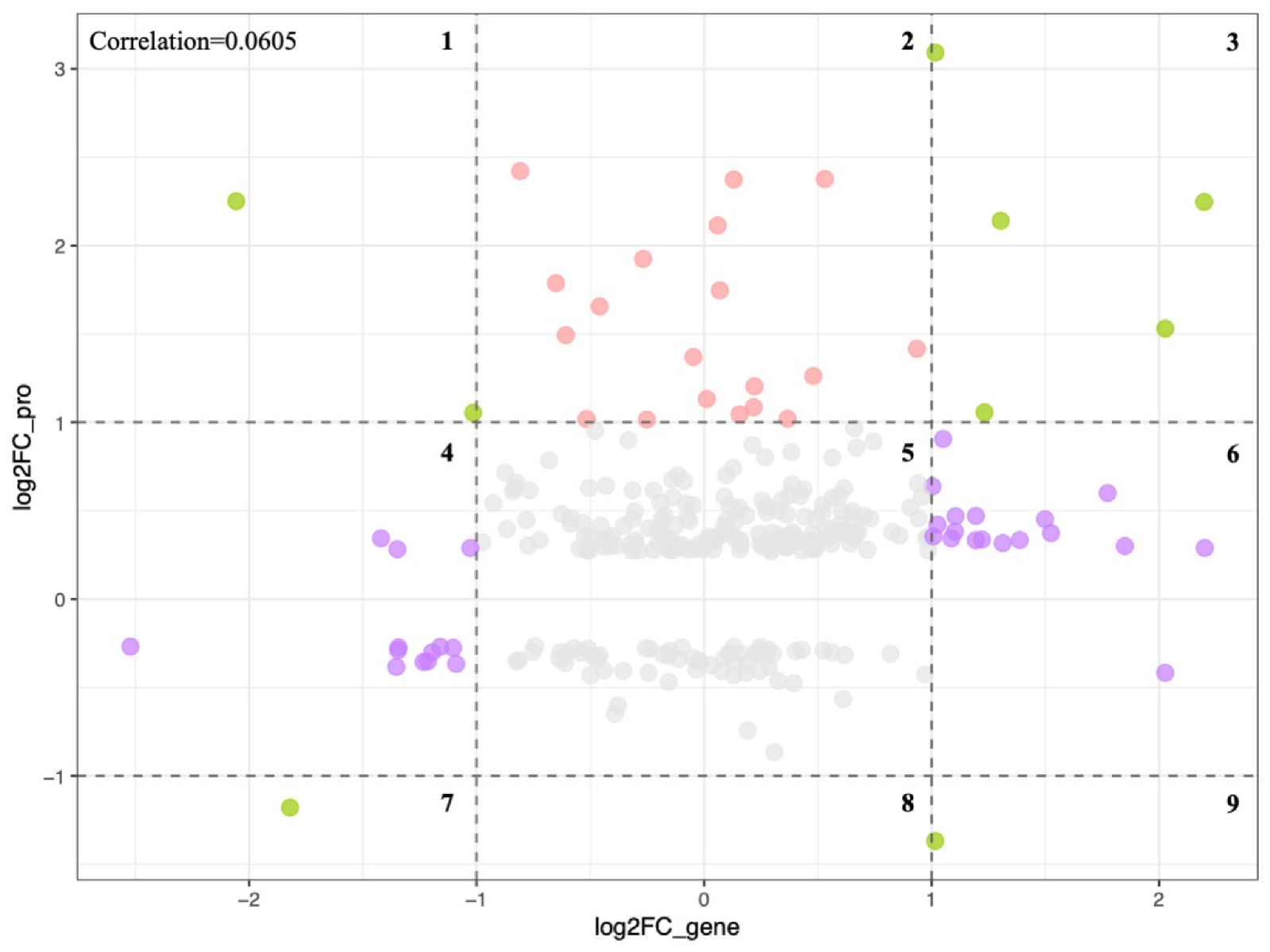

Supplement: Supplementary file 6 — Additional file 6: Figure S5. Correlation between transcriptomic and proteomic datasets for cocultured Synechococcus cscB+. The x-axis indicates the log2-fold change in genes, and the y-axis represents the log2-fold change in proteins. The genes with a 1.5-fold difference in transcriptome data were extracted, and the corresponding difference multiple and significance information (P-value) was obtained. The nine-quadrant diagram reflects the correlation between transcriptomic and proteomic datasets. Quadrant 5 indicates non-differentially expressed genes and proteins with multiple omics; the mRNA in quadrants 3 and 7 showed the same differentially expressed pattern with corresponding proteins; the protein expression abundance in quadrants 1, 2 and 4 represented posttranscriptional or translational level regulation; the protein expression abundance in quadrants 6, 8 and 9 was higher than that in mRNA. [file 13068_2022_2163_MOESM6_ESM.jpg]
